# Role of periosteum in alveolar bone regeneration comparing with collagen membrane in a buccal dehiscence model of dogs

**DOI:** 10.1038/s41598-023-28779-7

**Published:** 2023-02-13

**Authors:** Zhigui Ma, Ke Guo, Lu Chen, Xinwei Chen, Duohong Zou, Chi Yang

**Affiliations:** 1 Department of Oral Surgery, College of Stomatology, National Clinical Research Center for Oral Diseases, Shanghai Key Laboratory of Stomatology, Shanghai Research Institute of Stomatology, Shanghai Ninth People’s Hospital Affiliated to Shanghai Jiao Tong University School of Medicine, No. 639, Zhizaoju Road, Shanghai, 200001 People’s Republic of China; 2 Department of Stomatology, Tongren Hospital, Shanghai Jiao Tong University School of Medicine, Shanghai, People’s Republic of China

**Keywords:** Biomedical engineering, Animal disease models, Medical research

## Abstract

To investigate the role of periosteum on the treatment of buccal dehiscence defects comparing with collagen membrane in canine model. Bilateral dehiscence-type defects at the buccal side on the distal root of the lower 3rd/4th premolars were created in six beagle dogs with a total of 24 defects and assigned into three groups: Group A: blood clot in an untreated defect; Group B: deproteinized bovine bone material (DBBM) covered with an absorbable membrane; Group C: DBBM covered with the periosteum. The structural parameters for trabecular architecture and vertical bone regeneration were evaluated. Histological and histomorphometric evaluation were carried out to observe new bone formation and mineralization in the graft site. Immunohistochemical analysis was performed to identify the expression of osteopontin (OPN) and osteocalcin (OCN) at postoperative 3 months. Group C achieved greater vertical alveolar bone gain than that of group A and group B. The periosteum-covered group showed significantly greater new bone formation and accelerated mineralization. The greater immunolabeling for OPN and OCN was observed in group C than in group A. Periosteal coverage has explicit advantages over collagen membranes for the quality and quantity of new bone regeneration in dehiscence defects repairing.

## Introduction

The use of barrier membranes to facilitate new connective tissue attachment and enhance the osteogenic capability after bone grafting procedure has received wide acceptance. Several researches have manifested favorable healing results of the guided tissue regeneration (GTR) with barrier membranes for the treatment of periodontal defects^1–3^. These membranes used to prevent the rapid growth of connective tissue from migration to bone defects and to maintain space for the regeneration of alveolar bone and periodontal ligaments^4^. The periosteum has been considered a structure with remarkable regenerative capacity, which comprises of two layers. Osteoblast-like cells are located in the inner layer, which could facilitate bone formation and bone repairing without immunological reactions^5^. However, the periosteum is fairly difficult to obtain and has limited source on the body with injury on the second donor site. To avoid these disadvantages, collagen membranes have been explored in clinical practice.

Collagen membranes are absorbable barriers, which can elicit tissue reactions and surgery related complications as well as reduce patient discomfort for their biodegradable nature. Other clinical advantages of collagen membranes include excellent handling properties, clot formation and stabilization, hemostatic function, facilitating wound stabilization and healing, and the ability to recruit fibroblasts in vitro^6,7^. The application of this collagen membrane after surgical extraction of mesioangular or horizontally impacted wisdom teeth has stimulated bone regeneration and prevented periodontal defects^8^. Some authors also found that this type membrane could promote the attachment and proliferation of human periodontal ligament (PDL) fibroblasts and human osteoblast-like cells in vitro^4^. Although collagen membranes showed positive results and was widely used as bone graft scaffolds, the drawbacks including disease transmission, increased cost, host immune reactivity, fast biodegradation and limited osteogenic capability should not be neglected^9^.

As periosteum could be harvested in situ during GTR procedures, periosteal harnessing should be considered for the achievement of better therapeutic effects. Some researchers have investigated the healing ability of the critical-size bone defects in dogs with or without the preservation of periosteum. They found periosteum alone seems to be enough to permit spontaneous healing in mandibular defects. Spontaneous bone formation was limited in the case of periosteum absence^10^. Even some researches have focused on artificial periosteum from the perspective of structural and functional periosteal regeneration to repair the bone defect^11^. As a vital component of normal bone tissue, the periosteum could act as a barrier membrane during bone graft procedure and achieve favorable outcomes^12^.

To our knowledge, no comparative histomorphometric study regarding the resorbable collagen membrane and periosteum covering the grafts on a denuded root surface has been reported. The aim of this study was to histomorphometrically compare the role of commercially available collagen membrane (Bio-Gide^®^, Geistlich Biomateirals AG) and the periosteum on regeneration of surgically created buccal bony dehiscence defects in canine.

## Material and methods

### Animals

Six healthy male beagle dogs, aged 18–20 months (weighed 8.5–9.5 kg) were used and this study was approved by the institutional review committee of Shanghai Jiao Tong University School of Medicine (No. HKDL [2017] 368). The study was conducted in accordance with ARRIVE (Animal Research: Reporting In Vivo Experiments) guidelines for preclinical animal studies and the Guidelines laid down by the National Institute of Health (NIH) in the USA regarding the care and use of animals for experimental procedures. The animals were adapted to a 12-h light/12-h dark cycle for 1 weeks before the surgery. The dogs had free access to food and water in the entire experiment.

### Surgical management

**Figure 1:**
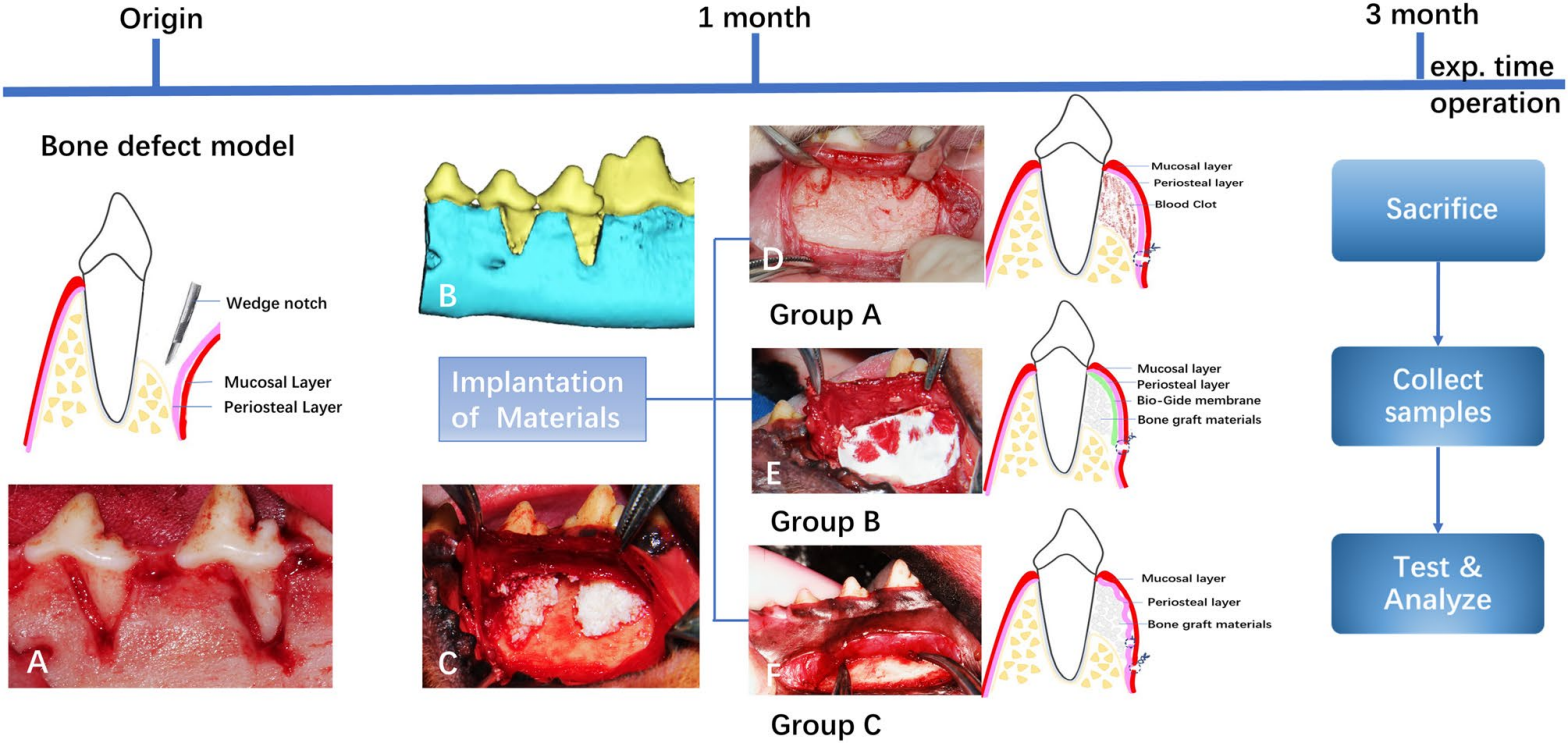
Repairing of buccal dehiscence defects in dogs. (**A**) Surgically created a buccal dehiscence model. (**B**) Reconstructed 3-D tomogram of dehiscence defect at postoperative 1 month. (**C**) subperiosteal placement of DBBM on the denuded root surface. (**D**) Blood clot filled in an untreated defect. (**E**) Bio-Gide collagen membrane coverage of the grafts. (**F**) Periosteum coverage of the grafts.

The experimental study was performed in two surgical phases. All the animals were anesthetized with 1.25% Sodium pentobarbital (0.4 ml/kg, IV) in the surgery. Buccal dehiscence type defects were created in the first phase. Six beagle dogs received dehiscence defects on the distal root of the mandibular 3rd, and 4th premolars (P3–P4) bilaterally (n = 4 defects per animal, n = 24 in total). Briefly, a full thickness mucoperiosteal flap was reflected, and standardized buccal dehiscence-type defects (6 mm in height from the cemento-enamel junction and 5 mm and 2 mm in width at the top and bottom, respectively) in each side of the lower jaw were created as described previously^13,14^. Root surfaces were denuded of periodontal ligament using a curette and then a mucoperiosteal flap was repositioned (Fig. S1, Supporting Information). After a 1-month healing period, this model could resemble buccal dehiscence defects confirmed by cone beam computed tomography (CBCT) (Fig. 1A–C and Fig.S2, Supporting Information). A total of 12 sides (each side contains the adjacent P3 and P4 of the lower jaw) were randomly assigned into the following treatment groups, including four sides each: Group A: blood clot in an untreated defect (n = 8, blank, n = 8); Group B: deproteinized bovine bone mineral with granule size of 0.25–1 mm and a volume of 0.25 ml per defect (DBBM, Bio-Oss, Geistlich Biomateirals AG, Wolhuser, Switzerland) was applied to bone regeneration, covered with a collagen barrier (n = 8,positive control, n = 8); Group C: the same amount of DBBM with the periosteum coverage (n = 8)^15^. In the second surgery, a horizontal incision was first performed at the mucogingival junction from the second premolar to the first molar without vertical releasing incisions for the group A. The mucoperiosteal flap was reflected coronally till near the gingival margin and then repositioned and sutured, only leaving blood clot in the defect area (Fig. S3, Supporting Information). Untreated defects served as the control group. For the group B, the operative approach was the same as group A, and Bio-Gide membrane were placed over the defect which was filled with DBBM (Fig. 1D, S4, Supporting Information). For the group C, care was taken not to cut the periosteum when a horizontal incision at the mucogingival junction was performed according to our previous clinical report^16^. To adequate exposure of the periosteum, a partial thickness flap separating the periosteum from the overlying mucosal layer was reflected coronally to the cervical portion and apically to the mental region (5 mm below the initial incision) using surgical scissors. This dissection was carefully performed to avoid laceration of the periosteum. The mental nerve and blood vessels should be protected with great cautions. Then the periosteum was incised at the base and then reflected coronally using a small periosteal elevator. Eventually, the semi-free periosteum layer as a nature barrier membrane was harvested with the similar dimension to the collagen membrane (Fig. 1E). The periosteum was repositioned and sutured after bone grafting with DBBM (Fig. 1F). Finally, the mucosal flap was repositioned and securely sutured (Fig. S5, Supporting Information). Penicillin (30,000 u/kg) was administered every day for 1 week and all animals were fed soft food with a plaque control regimen during the whole period of the experiment.

### Sequential fluorescent labeling

To evaluate the new bone formation and mineralization with time, the animals were injected intramuscularly with tetracycline (25 mg/kg, TE; Sigma, USA), alizarin red (30 mg/kg, AL; Sigma) and calcein (20 mg/kg, CA; Sigma) at postoperative 3, 6 and 9 weeks, respectively. The outcomes were analyzed as the reported methods^8^. Half of the samples were fixed for nondecalcified sectioning, dehydrated using ascending concentration of alcohols from 75 to 100%, infiltrated and embedded along the buccolingual plane in methyl methacrylate (MMA). Three serial buccolingual sections, with a thickness of 150 µm, were taken along the longitudinal direction of the teeth by a diamond-coated internal-hole saw microtome (Leica SP 1600, Milan, Italy), Each section was grounded and polished to a final thickness of 50–70 µm for fluorescent labeling observation a confocal laser scanning microscope (Leisa TCS, Germany). Sequential fluorochrome labeling for the newly formed mineralized bone was calculated according to the previous report^17^. The number of pixels labeled with yellow (TE), red (AL), and green (CA) in each image was measured as a percentage of the mineralization area, respectively, using an image analysis system (Image-Pro Plus software, Media Cybernetic, USA).

### CBCT and micro-CT evaluation

CBCT (Imaging Sciences International, Hatfield, PA, USA) was taken immediately and 1 month after operation for dehiscence creation in this study. Vertical alveolar bone loss (VABL) was defined as the distance between the crest of the alveolar bone and the cement-enamel junction in the long axis direction of distal root of P3 or P4 along the largest buccal-lingual section, which reflected effective periodontal bone support around the teeth.

To observe the internal structure of bone, micro-CT was performed for all animals that were sacrificed at 3 months post implantation. Blocks in the lower premolar regions, including bone, teeth and surrounding soft tissue, were harvested and fixed in 4% paraformaldehyde (PFA). The fixed samples were scanned using an animal Micro-CT scanner (mCT-80, Scanco Medical, Switzerland) to observe the newly formed bone at the buccal defects. The parameters of the micro-CT were set at 70 kV, 114 mA, 700 ms of integration time, a resolution of 2048 × 2048 pixels and an isotropic voxel size of 18 μm. The volume of interest was selected as the dehiscence defect and extended for a total of 500 slices. Bone volume to total volume ratio (BV/TV), trabecular number (Tb. N.) and bone mineral density (BMD) were analyzed^18^.

Because micro-CT can be cut freely and view from different perspectives, VABL was measured as the above method mentioned.

### Histological and histomorphometric observations

After fluorescence microscopy, the undecalcified sections were stained with Van Gieson’s picro-fuchsin. The percentages of newly formed bone and the residual bone substitutes were quantified at low magnification from three randomly selected sections from each specimen using image analysis system (Image-Pro-Plus, Media Cybernetic, USA). The other half of the blocks was decalcified with 10% of ethylenediaminetetraacetic acid (EDTA-2Na, pH  7.4) at 37 ℃ for 9 months and dehydrated in ethyl alcohol with gradually increasing concentrations from 70 to 100%. After embedding in paraffin, serial sagittal cross sections were made to a thickness of 4–5 µm. Three slices were chosen from each specimen. The first slice was from the long axis of distal part of teeth in a buccolingual direction and the rest were obtained 0.5 mm mesial and distal from the initial section. Sections were stained with hematoxylin and eosin (H–E) and images were captured by a light microscope for the observation of bone regeneration.

### Immunohistochemistry

For the detection of the bone remodeling proteins expression in the defect area, immunohistochemical analysis was performed using antibodies for osteopontin (OPN), and osteocalcin (OCN). The sections were treated with 3% H_2_O_2_ in methanol for 30 min and blocked endogenous peroxidase. And then these sections were incubated in Tris-buffered saline (TBS). Primary antibodies contained OPN (Rabbit; 1:100 dilution; Novus Biologicals, USA) and OCN (Rat; 1:100 dilution; Abcam, USA) were applied to the sections at 4 °C overnight. After three times washes with PBS, the slices were incubated for 30 min with biotiny-conjugated secondary antibody anti-rabbit or anti-rat IgG (Boster Bio Co. Ltd., Shanghai, China), and incubated with a preformed streptavidin biotin complex for 30 min. Staining was carried out by 3-3’-diaminobenzidine substrate (DAKO, Cambridge, UK), and the specimens were then counterstained with hematoxylin.

Protein expression levels were assessed by the mean optical density (MOD). Both the area and the integrated optical density (IOD) of positive stains were quantified with Image J software. MOD was calculated as follows: MOD = IOD/observed area^19,20^. The mean value was calculated and used as the final value.

### Statistical analysis

SPSS v.17.0 software (SPSS, Chicago, IL, USA) was used for statistical analysis. All the data are presented as mean ± standard deviation (SD). The differences among groups A, B and C were analyzed by analysis of variance. According to the data distribution and equal variance assumption test, Student–Newman–Keuls (SNK) post hoc or Friedman's test followed by Wilcoxon test for multiple comparisons were performed. All comparisons were conducted at the 0.05 level of significance.

## Results

All dogs recovered uneventfully with slight swelling within 1 week after surgery. No signs of wound inflammation or dehiscence were observed. In the Bio-Gide group, one dehiscence site did not show discernible sections and was excluded for histological observation.

### CBCT and micro-CT measurements

**Figure 2:**
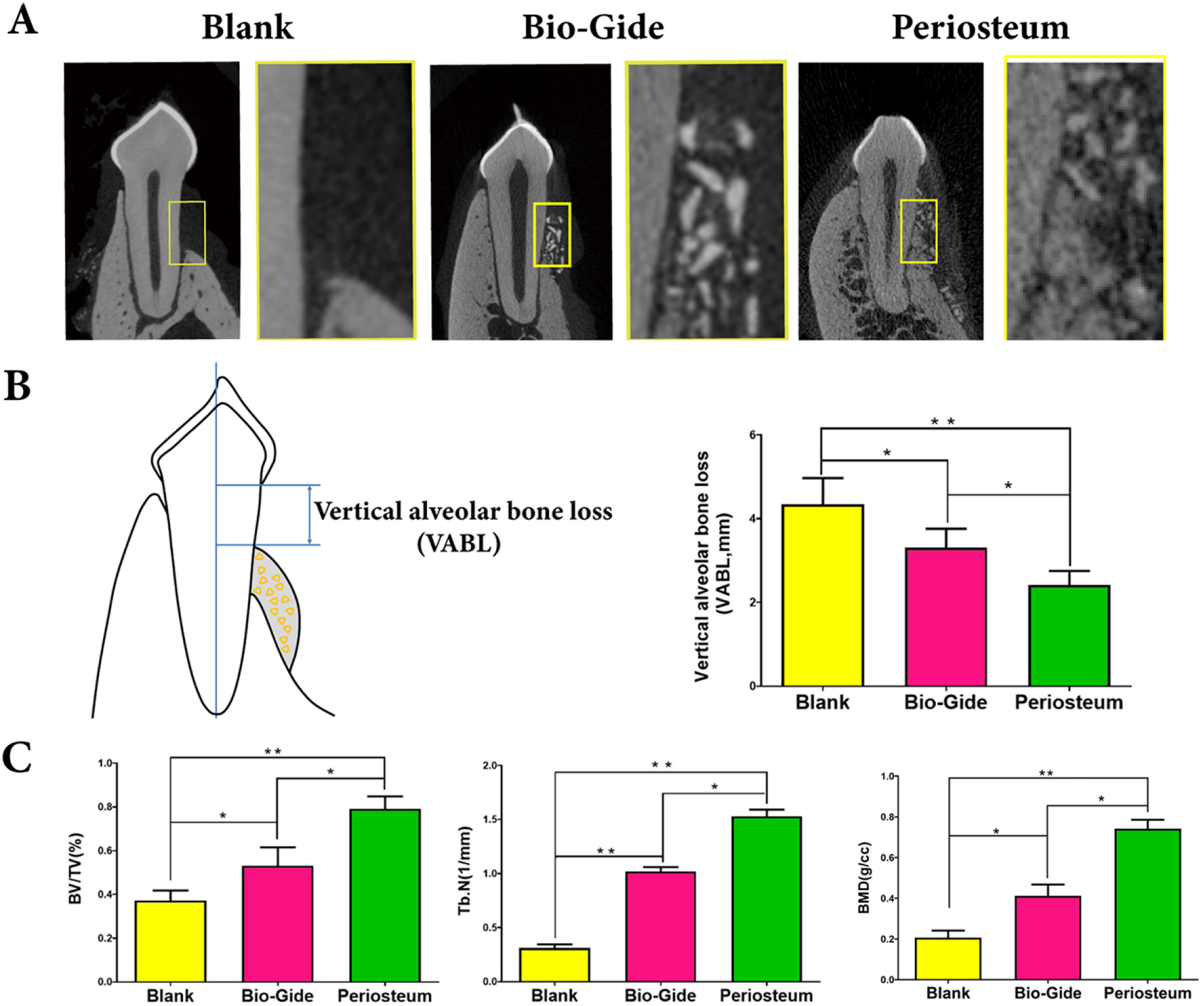
Micro-CT evaluation of buccal dehiscence repairing. (**A**) Representative sagittal images of buccal dehiscence defects 3 months after GTR procedure. (**B**) The schematic diagram of micro-CT assessments showing vertical alveolar bone loss (VABL) measured from the coronal aspect of new bone to the cement-enamel junction (CEJ) of the distal root of P3 or P4. (**C**) The histomorphometric analysis of bone volume/total volume (BV/TV), trabecular number (Tb. N) and bone mineral density (BMD). (*P < 0.05; **P < 0.01).

Combined with observation on CBCT and micro-CT, no statistically significant difference was found among three groups at initial or 1 month after defect creation. During the whole experiment period, there was also no obvious change found in the group A, which further confirmed the reliability for dehiscence defect model. Vertical augmentation of new bone in labial alveolar bone was successfully achieved in each treatment group and the periosteum had an advantage over the Bio-Gide membrane (Fig. 2A,B and Fig. S2, Supporting Information).

The micro-CT analysis showed that the bone volume/total volume (BV/TV) ratio was gradually increased from group A (37.28 ± 4.61%), group B (53.15 ± 8.75%) to group C (79.12 ± 5.73%) (*P* < 0.05). The trabecular number (Tb. N) showed the similar pattern as the BV/TV ratio. Additionally, the value of bone mineral density (BMD) in group C (0.74 ± 0.05 g/cc) was significantly higher than that in group B (0.41 ± 0.04 g/cc) (*P* < 0.05). All together, these results suggested that the periosteum could promote much more bone regeneration than Bio-Gide(*P* < 0.05) (Fig. 2C).

### Fluorochrome labeling analysis

**Figure 3:**
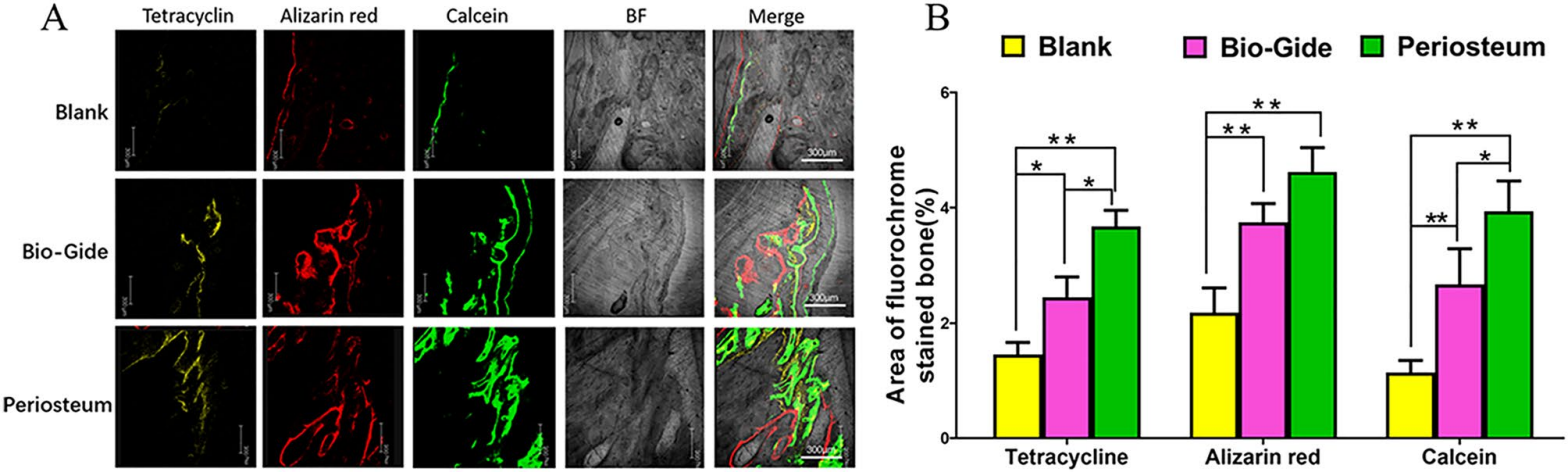
New bone formation and mineralization was determined histomorphometrically by tetracycline (TE), calcein (CA) and alizarin red (AL)- labeling analysis. (**A**) Confocal laser microscope images for the control, bio-Gide and periosteum groups, respectively. (**B**) The graph shows the percentage of each fluorochrome area for different groups. (*P < 0.05; **P < 0.01). The percentages of three fluorescent labeling in groups B and C were significantly higher than that in group A, and there were significant differences between the group B and group C in the percentage of TE and CA labeling (*P* < 0.05), which indicated that more newly formed bone was observed in the periosteum group.

Newly formed mineralized bone at three time points were evaluated by calculating different fluorescent labeling area. The tetracycline labeling percentage (yellow) was 1.45 ± 0.21%, 2.45 ± 0.35%, 3.67 ± 0.28% for the group A, group B and group C, respectively, with statistical significance (*P* < 0.05). The value of Alizarin red labeling percentage (red) in groups B and C was significantly higher than that in group A (*P* < 0.05). The percentage of the calcein labeling (green) had similar pattern as the tetracycline (*P* < 0.05) (Fig. 3). These data indicated that when the periosteum was applied to alveolar augmentation, new bone formation and mineralization was greater than that of the resorbable collagen membrane throughout the entire experimental period.

### Histological and histomorphometric evaluation

**Figure 4:**
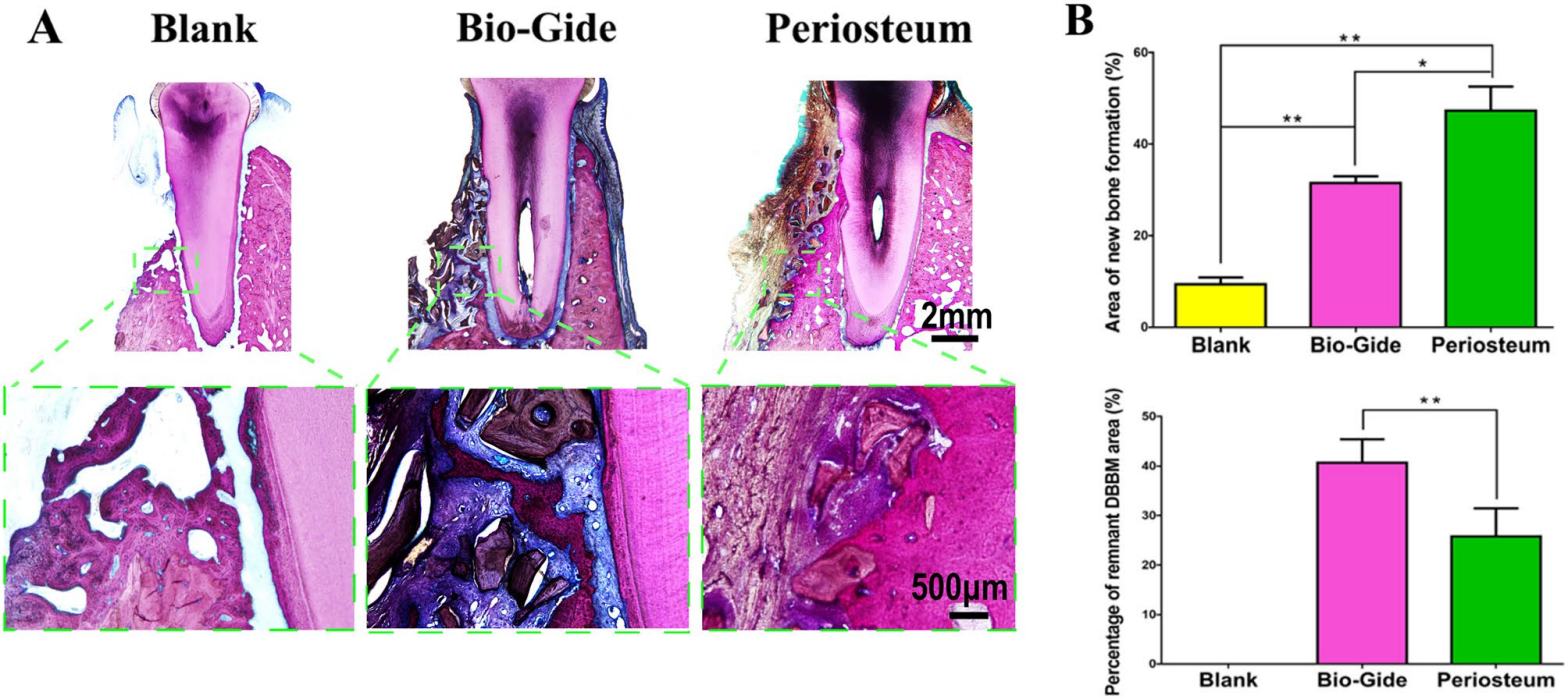
Histological analysis of the non-decalcified specimens. (**A**) Van Gieson's staining of the specimens in each group. The new bone was stained into red. The periosteum-covered group showed only limited residual DBBM encapsulated by fibrous tissue in the grafted area and higher new bone area than the other two groups. A small amount of new bone with much more DBBM remnants was observed in the Bio-Gide membrane-covered group, (**B**) Percentage of new bone area and residual DBBM area were assessed by histomorphometric measurement. The periosteum group showed significant higher new bone area and lower residual DBMM area than the Bio-Gide group, which demonstrated that the periosteum evidently accelerated the formation of new bone (*P < 0.05; **P < 0.01).

Based on the staining by Van Gieson method, the newly formed bone tissue was stained into red and showed a woven, trabecular appearance (Fig. 4A). A small amount of new bone extended from the bottom region of the defect was observed in the negative control group. Cementum and periodontal ligament were limited only in the apical portion of the defect. No ankylosis was observed. In group B, residual bone grafts were surrounded partly by the newly formed bone in the grafted region. Cementum-like tissue was formed on the apical root surface and appeared as a thin layer. In addition, periodontal ligaments were reestablished between the new formed cementum-like tissue and alveolar bone. In group C, considerable new alveolar bone occupied the periodontal defect area with some residual bone substitutes. Cementum-like tissue was relatively thick with a layered structure. PDL-like tissue formation was accelerated. Group C demonstrated the best outcome in vertical alveolar bone augmentation, followed by group B, and then group A. Furthermore, newly formed bone was greater in the apical regions of the specimens in comparison with the coronal regions. Overall, the percentages of new bone area at 3 months were 47.34 ± 5.24% in group C, 31.56 ± 1.44% in group B and 9.42 ± 1.45% in group A, with significant difference (*P* < 0.05). The percentages of remnant substitutes area were 40.64 ± 4.76% in group B, and 25.78 + 5.67% in group C, and the difference was statistically significant (Fig. 4B).

**Figure 5:**
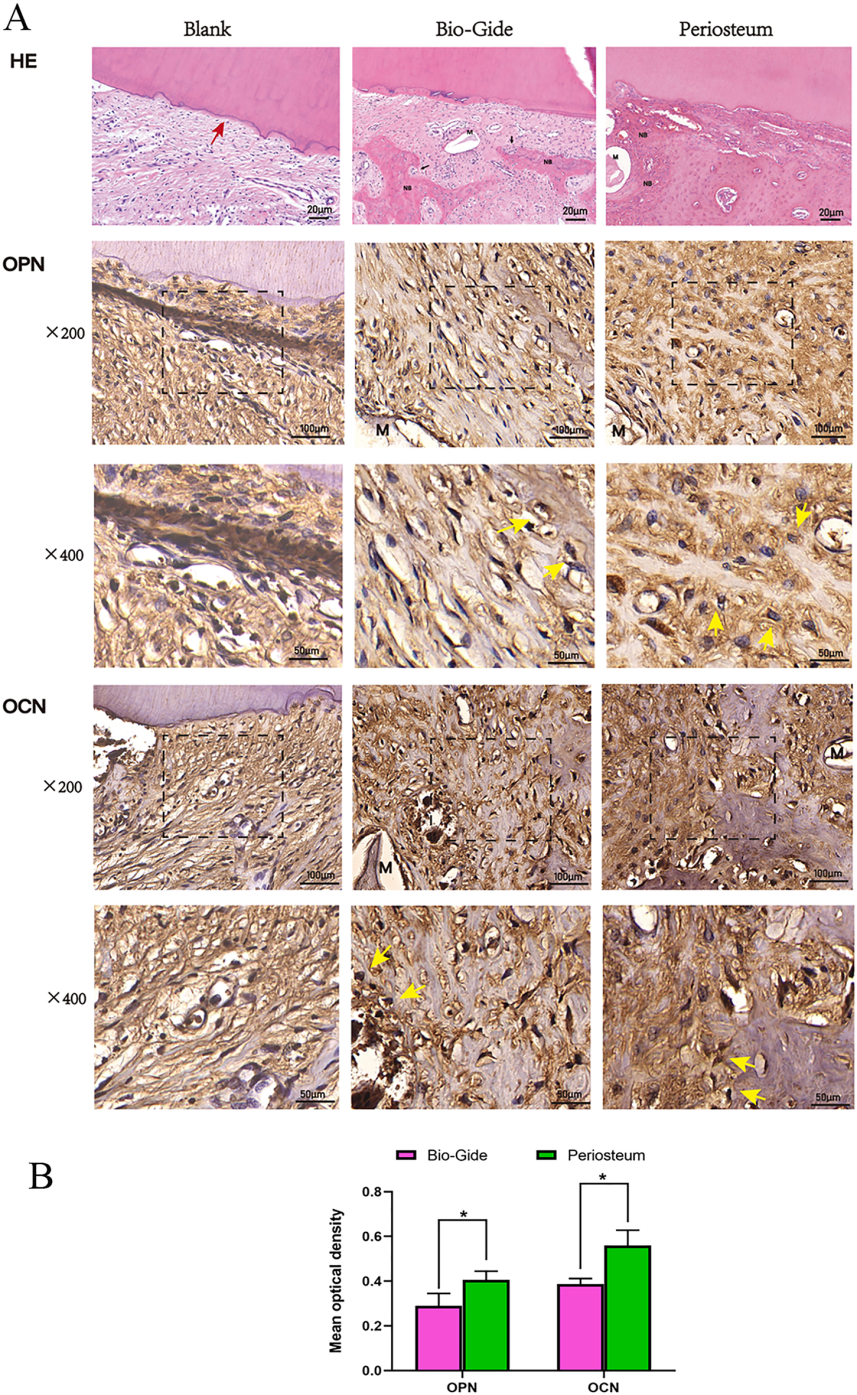
The results of H–E and immunohistochemical staining. (**A**) Decalcified H–E staining indicating the defect mostly occupied by the fibrous connective tissue in group A. a small amount of osteoid and immature woven bone in group B and newly formed osteoid bone fused with lamellar bone in group C. Representative immunohistochemical reactivity for OPN and OCN at 3 months post-GTR and more intense OPN and OCN staining was observed in group C than in the group B. (**B**) Immunohistochemical analysis (mean optical density, MOD value) of OPN and OCN in group B and group C. The amount of OPN and OCN expression was greater in group C than in group B (*P < 0.05). Red arrow shows the defect part on the root surface. Yellow arrows indicate the osteoblasts. *M* residual materials.

On histological analysis at 3 months after implantation, in the control group, without particulate grafts, the coronal portion healed with a long junctional epithelium and connective tissue attachment. Cementum-like tissue formation along the root surface was evident in the group B and C. Furthermore, blood vessels were distributed in the interface between the root surface and new alveolar bone in two grafted groups. In group B, a small amount of inflammatory cell infiltration with limited newly formed bone was observed in the defect region. However, significantly more new bone co-existed with the mature lamellar bone was seen in augmented area in group C when compared to the other two groups (Fig. 5A). In addition, Sharpey’s fibers inserting into both the new alveolar bone and cementum-like tissue suggested that functionally oriented periodontal ligament tissue was favorably re-established in periosteum-applied group.

Immunoreactivity of OPN and OCN appeared as a brownish color that was confined to the extracellular matrix and cytosolic compartments of osteoblasts and fibroblasts, which exhibited morphologies consistent with synthesis activity. The amount of OPN expression was greater in group C than in group B (MOD value: 0.29 ± 0.05 vs 0.41 ± 0.04, respectively, *P* < 0.05). Similarly, the expression of OCN in the group C was higher compared to the group B (MOD value: 0.39 ± 0.02 vs 0.56 ± 0.07, respectively, *P* < 0.05, Fig. 5).

## Discussion

This study investigated the role of periosteum and collagen membrane on bone regeneration of dehiscence-type defects model in beagle dogs. It was demonstrated that periosteum has explicit advantages for graft volume maintenance and tissue regeneration, comparing with collagen membrane as illustrated in Fig. 1.

Micro-CT imaging is considered the gold standard for bone morphology and micro-architecture. But it is a challenge for large animals to take micro-CT scanning in vivo multiple times over the course of the experimental period, thus CBCT that was widely applied in clinic was taken immediately and 1 month after post-creation in this study, respectively. The results of CBCT scans showed successful dehiscence creation in a canine model. Further, Micro CT demonstrated vertical augmentation of new bone in labial alveolar defect was successfully achieved in each treatment group, and the periosteum group was prior to the Bio-Gide membrane group.

Histologic evaluation of specimen obtained from periosteal group showed more new bone growth in this study, which were in conformity with previous animal experiments evaluating the contributions of periosteum to bone formation^21,22^. This is mainly attributed to inherent membrane properties. It is demonstrated that the periosteum has remarkable bone-regenerative capacity, serving as a source of osteoprogenitor cells in the cambium layer inside. The outer fibrous layer is highly vascularized and shows a microvascular network including endothelial pericytes with the capacity to differentiate into a large number of diverse cell types, containing osteoblasts^23^. The biophysical and chemical environment of periosteum-derived cells egressing from the inner layer of periosteum into the critical-sized defect modulates bone osteogenesis and healing^24^. The periosteal membrane is regarded as essential for successful bone remodeling and formation to bridge a massive defect in a long bone^25^. The osseous structures and vascular circumstances in the mandible are similar to those in the long bone^26^. Moreover, it seems more important for the mandible, in which the generable blood supply is less anastomotic and diffuse than that in the maxilla^27^.

OPN protein that is distributed throughout the entire mineralized matrix is an important indicator for primary bone formation assessment. Concerning the immunohistochemical results, the periosteum group exhibited a greater number of OPN-positive osteoblasts. Much stronger immunolabeling for OCN, a bone formation biomarker for osteoblasts during the later stage of bone remodeling, was observed at 3 months post-implantation. These findings showed intense activity of alveolar bone formation, suggesting that the periosteum can accelerate the bone repairing processes.

The periosteum was considered as a "membrane" or boundary template for GTR. Therefore, several clinical procedures have been developed to utilize the bone-regenerative capability of the periosteum. Some studies have shown that a split-thickness flap and lateral replacement of the periosteum can be used as a barrier to stimulate bone formation in periradicular surgery^28^. Similarly, in the present study, periosteal harnessing provides primary tension-free flap closure via splitting of the mucosa, which should be viewed as a key step for the achievement of better outcomes in bone augmentation procedures.

The collagen membrane is traditionally considered as a physical barrier in GTR, which can prevent the undesirable invasion of surrounding connective tissues into the bone graft area and provide a stable environment for bone growth during healing^29^. Early removal of barrier membranes could lead to less bone formation or insufficient bone filling^30^. It is suggested that membranes should last for 3–9 months for GTR. Bio-Gide might undergo fast degradation in 4–8 weeks which could result in loss of structural integrity and a chronic inflammatory infiltrating around the membrane^31^. Long lasting membranes are still under investigation to fulfill this goal. However, the autogenous periosteum has particular advantage, as this barrier membrane is biocompatible, easy to harvest and manipulate. Further, vascular supply is also ensured due to in-situ periosteal coverage. Thus, the autogenous vascularized periosteum could be considered an effective alternation to the existing barrier membranes. In addition, previous research have demonstrated that bone grafts, used as space maintainer, in association with collage membranes not only provide compensation for the lack of stiffness of the membranes, but also lead to an increase in bone formation at the recipient site in comparison with using membrane alone^32^. Thus, DBBM was used in two grafted groups in the present study to support the membrane preserving its original position.

The results of the specimens for both membrane-covered groups exhibited much more new bone was regenerated in the apical regions as compared with the coronal regions at 12-week follow-up. One explanation is that the membranes in this study could not help maintain enough spatial stability and therefore lead to graft materials collapse. What is worse, a downward sloping contour of the mandibular alveolus could not help to prevent bone grafting from displacement, which may lead to insufficient bone regeneration at the coronal aspect. However, supracrestal bone regeneration is essential for the dehiscence treatment. Lack of enough space maintenance ability would result in the reduction of new bone formation^17^. In the present study, concerning views mentioned above, stable space environment may serve for the advantages of periosteal for graft volume maintenance and tissue regeneration, which may provide a new prospective to reconsider the core factor contribute tissue regeneration for repairing buccal dehiscence-type defects. The prospect of this membrane may decrease micro movement and improve its stabilization, thus enhanced its protective effects in the sub-membranous augmented area and eventually facilitated bone regeneration at the desired position, which needs further investigation. GTR membranes should be fabricated with great precision to facilitate structural and functional tissue regeneration. In the future, with computer-aided design (CAD) and 3-D printing technology, the regenerative membrane with more personalized designing could be used to manage irregular periodontal defects.

There are still several limitations in this study. The present defect model could not completely represent the real clinical situation. So far, it remains unconfirmed whether periosteum loss with dehiscence in the clinical, and only human histology can get reliable information. In addition, based on the present results, the underlying mechanisms of the periosteum on bone regeneration need to be clarified in future studies. Addition to above, although multiple comparison was selected before, we did not calibrate it so as not to miss any meaningful results.

## Conclusion

Our results demonstrated that significant bone regeneration can be achieved with the applied of periosteum to cover the grafts than the bioresorbable membrane, indicating the potential for this material to support better therapeutic effects during regenerative procedures for dehiscence. The underlying mechanisms of bone regeneration provided by periosteum is needed to be investigated in the future studies.

## Supplementary Information

Supplementary Figures.

## Data Availability

All data generated or analyzed during this study are included in this published article (and its supplementary information files).
