# Supplementary material for: Role of periosteum in alveolar bone regeneration comparing with collagen membrane in a buccal dehiscence model of dogs
Source: Sci Rep. 2023 Feb 13;13:2505. doi: 10.1038/s41598-023-28779-7 (PMC9925434; doi:10.1038/s41598-023-28779-7)
Supplement: Supplementary file 1 — Supplementary Figures. [file 41598_2023_28779_MOESM1_ESM.docx]

APPENDIX A. SUPPLEMENTARY DATA

**Role of** **periosteum in** **alveolar bone regeneration comparing with collagen membrane in beagle dogs**

**Role of periosteum in alveolar bone regeneration comparing with collagen membrane in beagle dogs**

Zhigui Ma^1*^ Ph.D., Ke Guo^2*^ M.S., Lu Chen^1^ M.S., Xinwei Chen^1^ Ph.D., Duohong Zou^1#^ Ph.D., Chi Yang^1#^ Ph.D.

^1^ *Department of Oral Surgery, Shanghai Ninth People’s Hospital, College of Stomatology, Shanghai Jiao tong University School of medicine; National Clinical Research Center for Oral Diseases; Shanghai Key Laboratory of Stomatology & Shanghai Research Institute of Stomatology. Shanghai, P.R.China*

^2^ *Department of Stomatology, Tongren Hospital, Shanghai Jiao Tong University School of Medicine. Shanghai, P.R.China*

Zhigui Ma, Associated professor, Department of Oral Surgery, Shanghai Ninth People’s Hospital affiliated to Shanghai Jiao tong University, school of medicine, Shanghai Key Laboratory of Stomatology. Shanghai, P.R.China.

Ke Guo, Resident, Department of Stomatology, Tongren Hospital, Shanghai Jiao Tong University School of Medicine. Shanghai, P.R.China.

Lu Chen, Laboratory technicians, Department of Oral Surgery, Shanghai Ninth People’s Hospital affiliated to Shanghai Jiao tong University, school of medicine, Shanghai Key Laboratory of Stomatology. Shanghai, P.R.China.

Xinwei Chen, Resident, Department of Oral Surgery, Shanghai Ninth People’s Hospital affiliated to Shanghai Jiao tong University, school of medicine, Shanghai Key Laboratory of Stomatology. Shanghai, P.R.China.

Duohong Zou, Professor, Department of Oral Surgery, Shanghai Ninth People’s Hospital affiliated to Shanghai Jiao tong University, school of medicine, Shanghai Key Laboratory of Stomatology. Shanghai, P.R.China.

Chi Yang, Professor, Department of Oral Surgery, Shanghai Ninth People’s Hospital affiliated to Shanghai Jiao tong University, school of medicine, Shanghai Key Laboratory of Stomatology. Shanghai, P.R.China.

****Dr. Zhigui Ma and Dr. Ke Guo contributed equally to this work and should be considered co-first authors.***

**^#^Corresponding Authors:**

D. Zou, Department of Oral Surgery, Ninth People’s Hospital, Shanghai Jiao Tong University, School of Medicine, Shanghai Key Laboratory of Stomatology, National Clinical Research Center of Stomatology, No. 639, Zhizaoju Road, Shanghai 200001, China. Email: zouduohongyy@126.com

C. Yang, Department of Oral Surgery, Ninth People’s Hospital, Shanghai Jiao Tong University, School of Medicine, Shanghai Key Laboratory of Stomatology, National Clinical Research Center of Stomatology, No. 639, Zhizaoju Road, Shanghai 200001, China. Email: yangchi1963@hotmail.com

**Supplemental Figures**


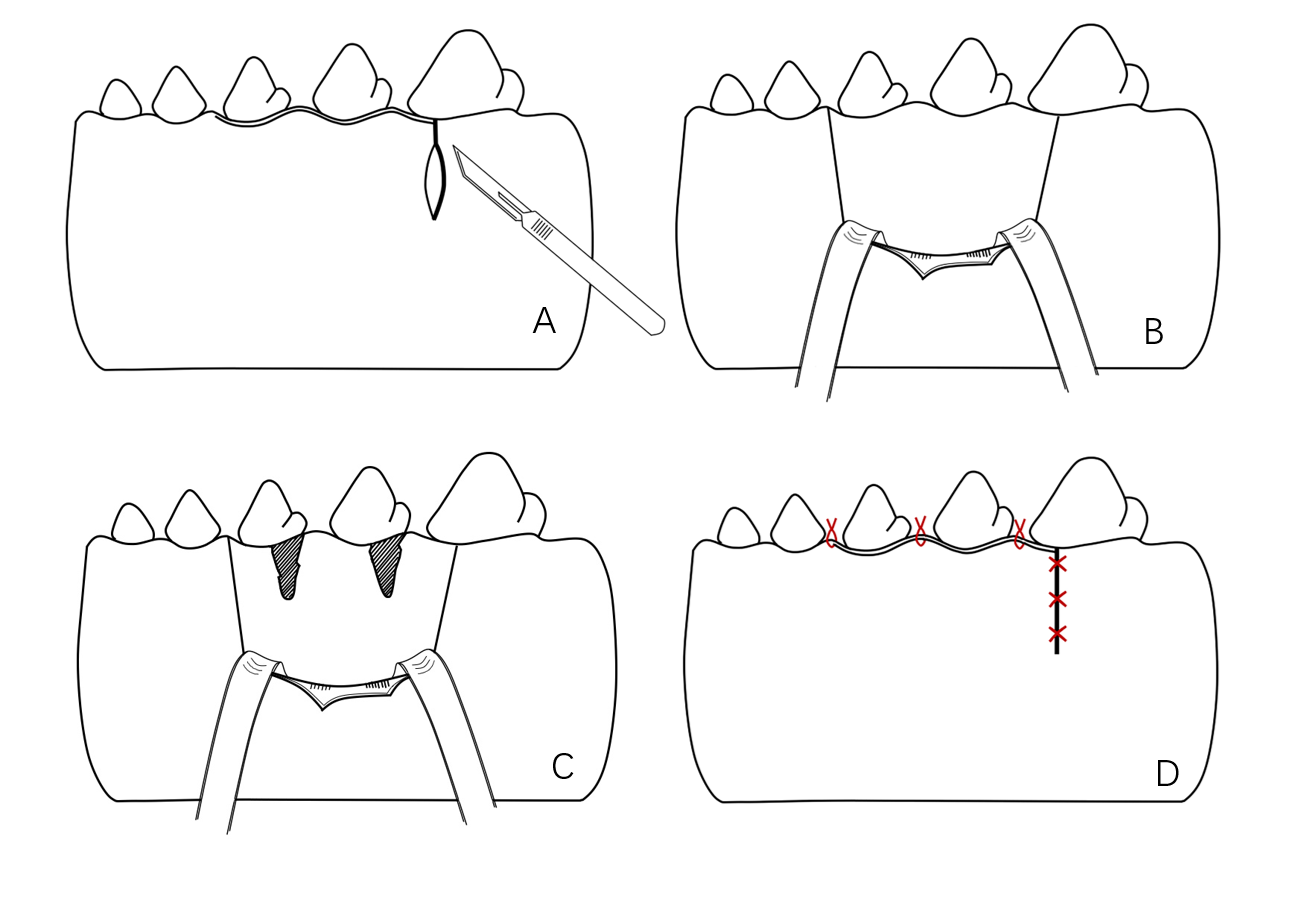


Fig.S1 Procedure for creation of buccal dehiscence defect. (A) A full thickness mucoperiosteal incision from the gingival sulcus. (B) Reflecting the mucoperiosteal ﬂap. (C) Creation of standardized dehiscence-type defects. (D) Repositioning the flap and closing the wound.


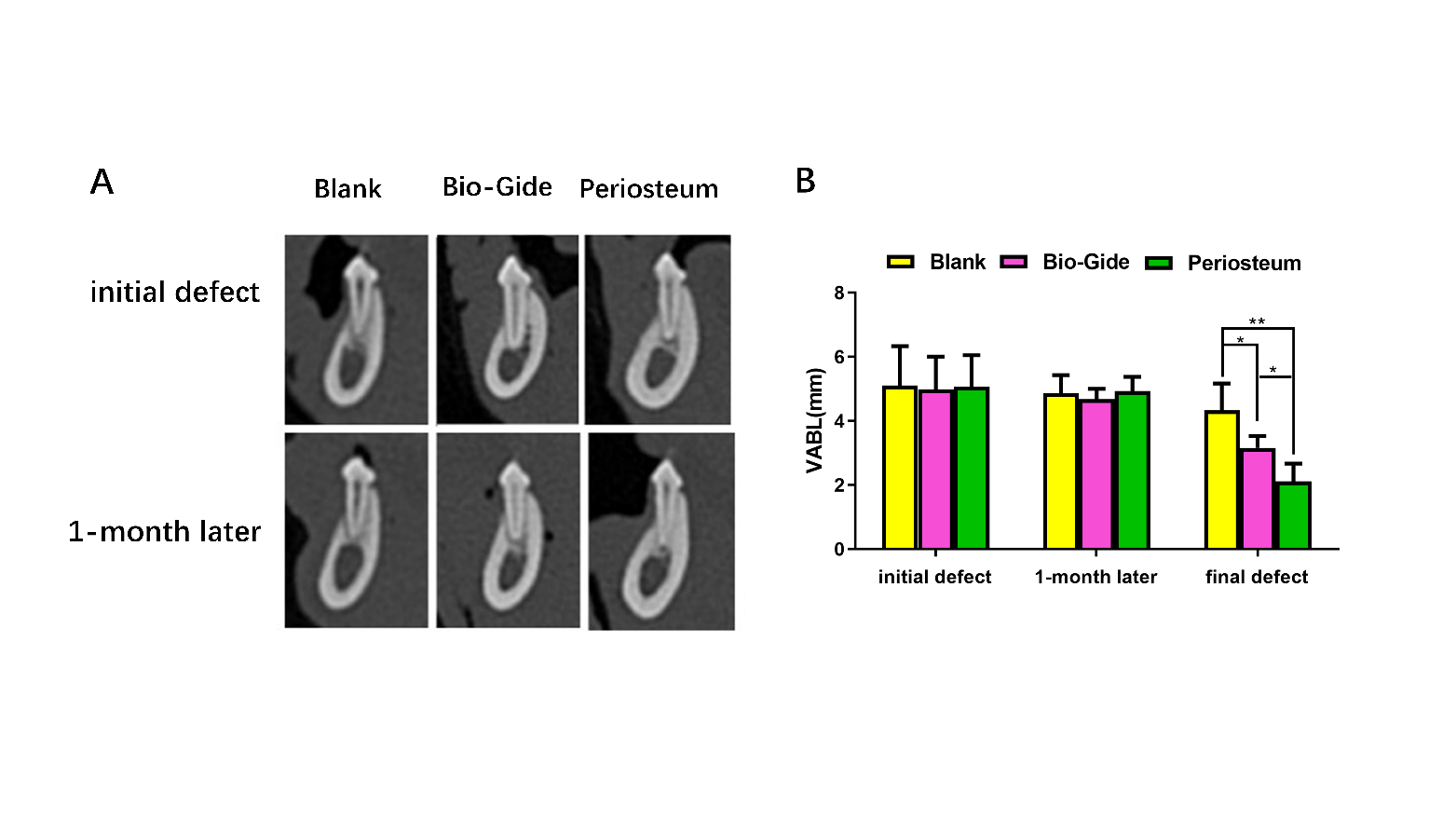


Fig.S2 Verification of buccal dehiscence defect model. (A) CBCT scans were performed at initial dehiscence creation and 1 month later for 3 groups. (B) No significant difference in vertical alveolar bone level (VABL, from CEJ to the base of the defect in the largest bucco-lingual section) was found between these three groups or within groups at the initial and 1 month later. Significant differences were observed among 3 groups (**P*<0.05, ** *P*<0.01).


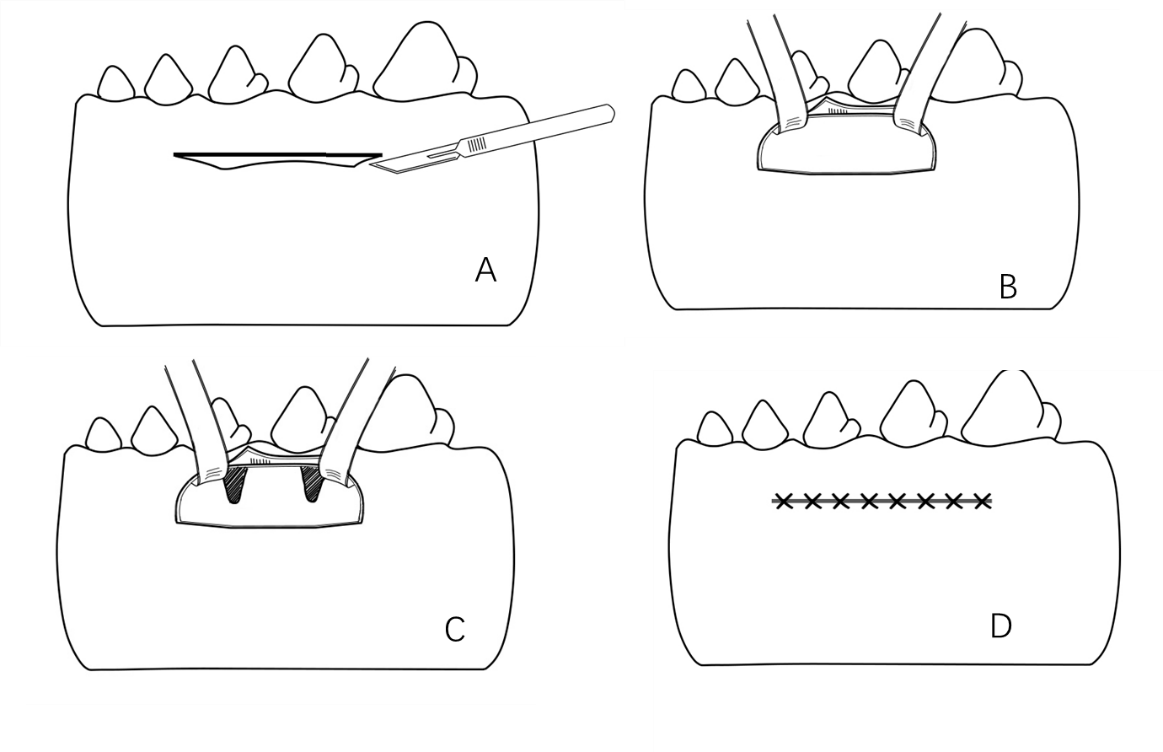


Fig. S3 Procedure for negative control group 1-month after creation of dehiscence-type defects. (A) A full thickness mucoperiosteal incision at the mucogingival junction (M1). (B) Coronal reflecting the mucoperiosteal ﬂap. (C) Blood clot in the defect area. D, repositioning the flap and closing the wound.


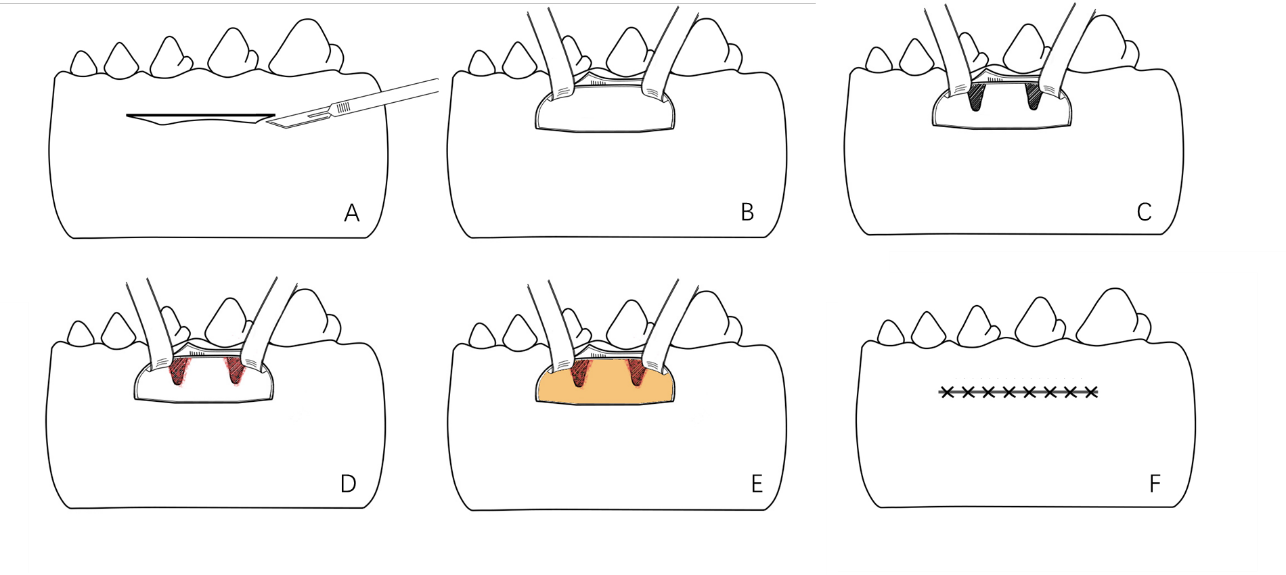


Fig.S4 Procedure for Bio-Gide membrane covered group. (A-C) The operative approach was the same as negative control group. (D)The defect was filled with DBBM. (E) Bio-Gide membrane were placed over the defect to ensure the stability of the grafted materials. (F) Repositioning the flap and closing the wound.


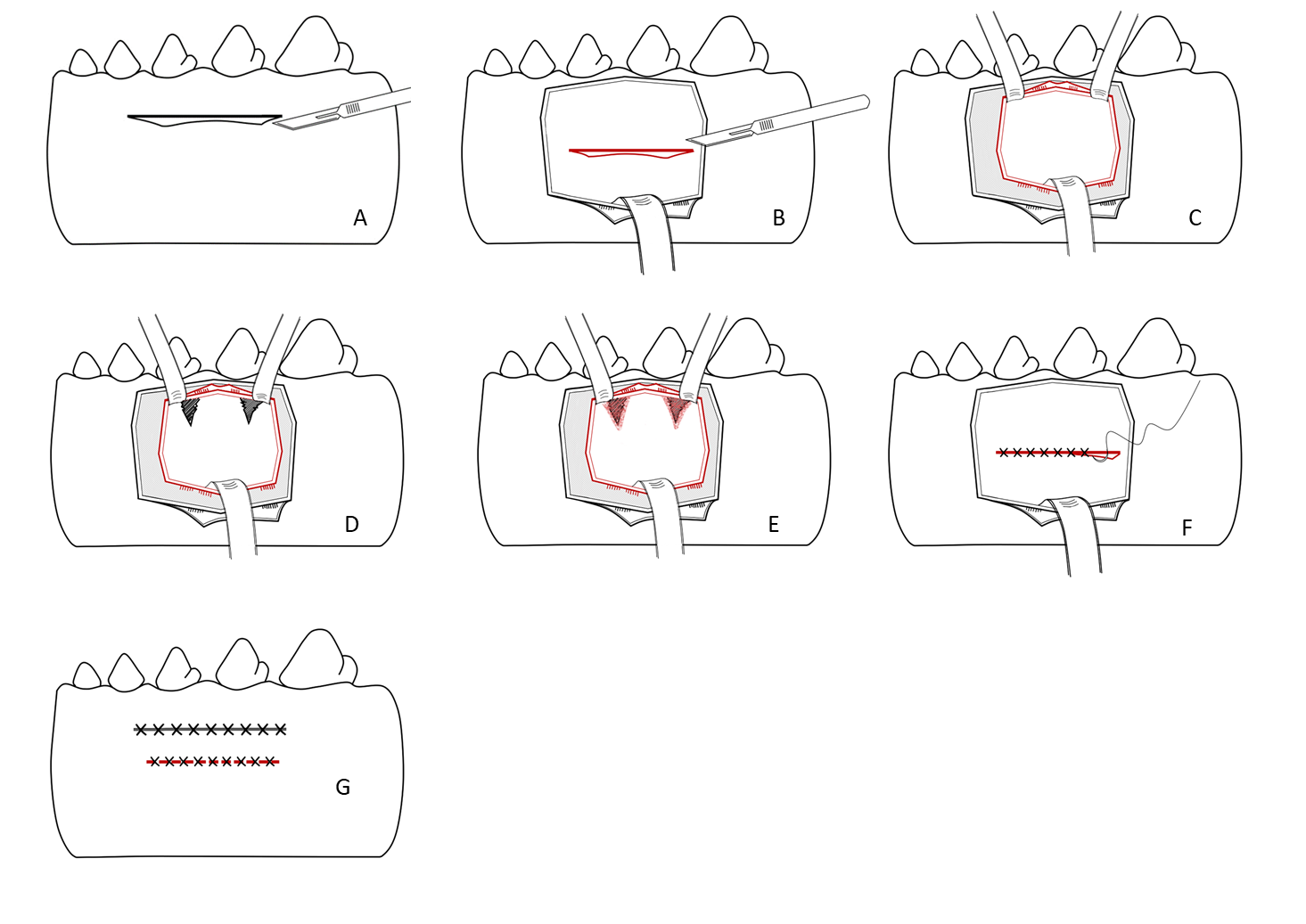


Fig. S5 Procedure for periosteum covered group. (A) A horizontal incision at the mucogingival junction was first made from mesial P3 to distal P4 without vertical releasing incisions. (B) A partial full-thickness flap was reflected apically by sharp dissection to ensure the operation remaining on the periosteal surface. (C)The periosteum was incised 5mm below the initial incision and reflected coronally so that the periosteum dimension was similar to that of the collagen membrane. (D)Exposure of the defect area. (E) Bone grafting with DBBM over the defect area. (F) The periosteum was repositioned and sutured. (G) Repositioning the flap and closing the wound. Black outline in the surgical area represents the mucosal flap and red color represents the periosteum.
